# Effect of Severe Plastic Deformation on Structure and Properties of Al-Sc-Ta and Al-Sc-Ti Alloys

**DOI:** 10.1186/s11671-017-1995-y

**Published:** 2017-03-23

**Authors:** Alla Berezina, Tetiana Monastyrska, Olexandr Davydenko, Oleh Molebny, Sergey Polishchuk

**Affiliations:** 1 G.V. Kurdyumov Institute for Metal Physics of National Academy of Sciences of Ukraine, 36, Academician Vernadsky Blvd., Kyiv-142, Kyiv, 03680 Ukraine; 2 O.O. Galkin Donetsk Institute for Physics and Engineering of National Academy of Sciences of Ukraine, 46, Prospect Nauki, Kyiv, 03039 Ukraine

**Keywords:** Al-Sc alloy, Severe plastic deformation, Hydrostatic extrusion, Equal-channel angular hydroextrusion, Dynamic recrystallization, Disclinations, Deformation bands, Low-angle and high-angle boundaries, Aging, Supersaturated solid solution, 81. Materials science, 61.66.Dk Alloys, 61.72.Ff Direct observation of dislocations and other defects (etch pits, decoration, electron microscopy x-ray topography, etc.)

## Abstract

The comparative analysis of the effect of monotonous and non-monotonous severe plastic deformations (SPD) on the structure and properties of aluminum alloys has been carried out. Conventional hydrostatic extrusion (HE) with a constant deformation direction and equal-channel angular hydroextrusion (ECAH) with an abrupt change in the deformation direction were chosen for the cases of monotonous and non-monotonous SPD, respectively. Model cast hypoeutectic Al-0.3%Sc alloys and hypereutectic Al-0.6%Sc alloys with Ta and Ti additives were chosen for studying. It was demonstrated that SPD of the alloys resulted in the segregation of the material into active and inactive zones which formed a banded structure. The active zones were shown to be bands of localized plastic deformation. The distance between zones was found to be independent of the accumulated strain degree and was in the range of 0.6–1 μm. Dynamic recrystallization in the active zones was observed using TEM. The dynamic recrystallization was accompanied by the formation of disclinations, deformation bands, low-angle, and high-angle boundaries, i.e., rotational deformation modes developed. The dynamic recrystallization was more intense during the non-monotonous deformation as compared with the monotonous one, which was confirmed by the reduction of texture degree in the materials after *ECAH*.

## Background

The study of severe plastic deformations in the recent two decades has led to the important conclusion that the plastic flow of a material occurs heterogeneously at any stage and at any level [1–3]. Experimental studies of the localization of the plastic flow carried out in the late XX century confirmed the multiple forms of this phenomenon [3–6]. It was shown that the macrolocalization of the deformation was typical of any material and any loading conditions. Localization forms of the deformation strictly conformed to the rule of correspondence between the distributions of macrolocalization centers, their kinetics and the stag of the loading curve [3].

The study of the effect of severe plastic deformation (SPD) on the structure and properties of the alloys showed that the use of strong plastic deformation even at room temperature causes processes of dynamic recrystallization and helps produce a fine-grained crystalline structure. Various methods of SPD, multiple rolling, multiple drawing, torsion under pressure, and equal-channel angular pressing (ECAP), were proposed [7–9]. ECAP allows inducing strong deformation in the material without changing sizes and, therefore, is of particular interest. Studies of the effect of severe plastic deformation on the structure and properties of alloys were focused, primarily, on determining the conditions of the formation of submicron and nanograin structures [9]. The processes of phase transformations and thermal stability of the metastable states appearing during severe plastic deformation were not studied adequately.

The aim of the study was to examine the effect of severe plastic deformation on the decomposition of supersaturated solid solutions to obtain an additive contribution to strengthening due to the fine-grained matrix and nanoscale strengthening phase formed during aging. Comparative analysis of using monotonous and non-monotonous severe plastic deformations of aluminum alloys was carried out.

## Methods

Conventional hydrostatic extrusion (HE) with a constant deformation direction and equal-channel angular hydroextrusion (ECAH) with an abrupt change in the deformation direction were chosen for the cases of monotonous and non-monotonous SPD, respectively. The method of equal-channel angular hydroextrusion, first developed at the Donetsk Institute for Physics and Engineering, NAS of Ukraine [10], is a modified method of equal-channel angular pressing.

Cylindrical billets were extruded from the container by a high-pressure fluid through an angular die with the angle *Φ* = 90°. Deformation was produced at room temperature under the pressure of 150 MPa for HE and 700 MPa for ECAH. The value of the accumulated equivalent strain during ECAH was approximately evaluated as ${e}_N=\frac{2 N}{\sqrt{3}} c t g\pi$, where *N* is the number of strain cycles, *Ф* is the angle of the intersection of channel segments. Model cast hypoeutectic Al-0.3%Sc alloys and hypereutectic Al-0.6%Sc alloys with Ta and Ti additives were chosen for the study.

The studied materials and deformation modes are given in Table 1.

**Table 1:** The compositions of the studied alloys and deformation modes

Alloy	Specimen	HE *e*	ECAH		HE *e*	Accumulated strain *e*_Σ_	*H*_*V*_
			Number of passes, *n*	*e*			
Al-0.3%Sc	1	0.23	5	5.8	0.77	6.8	48.9
Al-0.6%Sc-0.05%Ta	2	–	–	–	0.74	0.74	–
	3	–	1	1.16	0.74	1.39	–
Al-0.6%Sc-0.2%Ta	4	0.23	5	5.8	0.77	6.8	50.2
Al-0.6%Sc-0.6%Ta	5	0.23	5	5.8	0.77	6.8	52.8
Al-0.6%Sc-0.3%Ta	6	0.47	2	2.3	–	2.77	46.92
	7	2.01	–	–	–	2.01	51.08
	8	2.82	–	–	–	2.82	50.83
	9	0.47	4	4.6	–	5.07	50.83
	10	0.47	4	4.6	1.46	6.53	53.7
Al-0.6%Sc-0.1%Ti	11	0.47	4	4.6	–	5	63.83
	12	0.47	4	4.6	1.61	6.61	63.08
	13	0.47	4	4.6	2.42	7.42	62.75
Al-0.6%Sc-0.3%Ti	14	0.47	3	3.45	–	3.92	56.6

The structures of the alloys in the initial state after SPD and aging were studied using transmission electron microscopy (JEM-2000FXII), metallography, and Vickers hardness measurements. The temperature intervals of the phase transformations were determined by measuring the temperature coefficient of resistivity *α*_*t*_ *= 1/ρ*_*0*_ *dρ/dT.*

The X-ray analysis of deformed specimens was conducted using DRON-4M diffractometer in the Co-Kα radiation. A polycrystalline SiO_2_ specimen was used as a standard.

Parameters of the specimen microstructure were determined using X-ray diffraction peak profile analysis by the approximation method through Voigt function, which was the convolution of the Cauchy and Gauss functions [11]. The coherent domain sizes and lattice microdistortions in the specimens were estimated by the analysis of diffraction patterns using the classical Williamson-Hall method. The texture analysis was conducted using X-ray DRON-3 diffractometer with a texture attachment in Co-Kα radiation. The measurement was carried out using parallel beam geometry and scan angles from 0 to 80° and from 0 to 360° for *α* and *β*, respectively. Data collected on a textureless BaTiO_3_ specimen were used to take into account the defocusing effect.

The analysis of the crystallographic texture was carried out by constructing pole figures (PFs) using MTEX Matlab software package [12].

## Results and Discussion

### Characteristics of the Initial State of the Alloys

Hypoeutectic Al-0.3% Sc alloy crystallized through cellular mechanism. The grain size was about 2 mm. Al_3_Sc particles of 1–2 μm size were present at cell boundaries. The equilibrium volume fraction of Al_3_Sc phase was 0.2%.

**Fig. 1:**
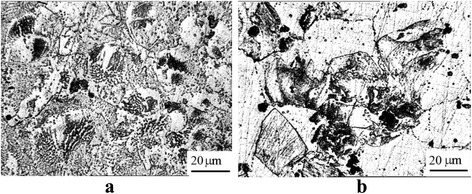
Al-0.6%Sc-0.05%Ta alloy: eutectic structure in the cast state (**a**), grain structure after hydroextrusion (**b**)

Hypereutectic Al-0.6%Sc-TM alloys crystallized through a combined mechanisms eutectic and peritectic. The equilibrium volume fraction of the Al_3_Sc phase was 0.9%. The grains of 150–250 μm size crystallized through the eutectic mechanism (Fig. 1a). Eutectic was a “fan structure” whose branches were the particles of semicoherent Al_3_Sc phase of 0.5–1.5 μm size.

Fine grains of about 30–60 μm crystallized through the peritectic mechanism and contained large particles of ~10 μm in their centers. The mixed structure of the Al-0.6%Sc-0.05%Ta alloy persisted after HE (*e* = 0.23) (Fig. 1b).

### The Structure of the Cast Alloys After SPD by Hydrostatic Extrusion and Equal-Channel Angular Hydroextrusion

Alternating bands with different reflectivities were observed after SPD by metallographic studies using electro-polishing of sections. Lamination of the material into two structural states was observed: the dark range—the range of localized deformation, and the bright one—the range free from stress. These ranges formed the unique domains of localized plastic deformation.

**Fig. 2:**
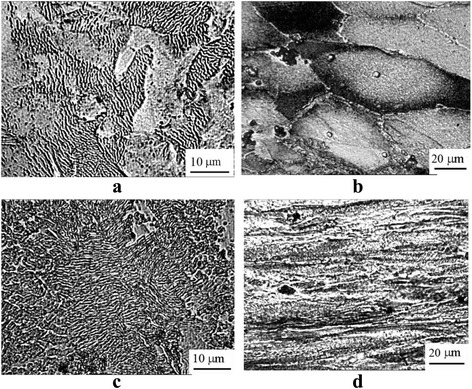
The structure of Al-0.6%Sc-0.05%Ta alloy after deformation: HE, *e* = 0.74, cross-section (**a**); longitudinal section (**b**); 1ECAH + HE, *e*_Σ_ = 1.9, cross-section (**c**); longitudinal section (**d**)

In the cross section, domains of localized deformation after HE (*e* = 0.74) were arranged irregularly (Fig. 2a). They nucleated heterogeneously at grain boundaries. The modulation period of domains was ~0.8–1 μm. Single bands of localized deformation could be observed in the matrix where the domains were absent. In the longitudinal section of the samples, domains of the localized deformation were absent; grains of ~130 × 40 μm size were elongated in the direction of extrusion. Particles of Al_3_Sc phase of ~2–4 μm size were present inside the grains.

The modulation period was reduced to ~ 0.5 μm after increasing the accumulated strain to *e*_Σ_ = 1.9 by using one pass of ECAH (Fig. 2c). In the longitudinal section, knife-like grains ~0.5 μm thick emerged.

**Fig. 3:**
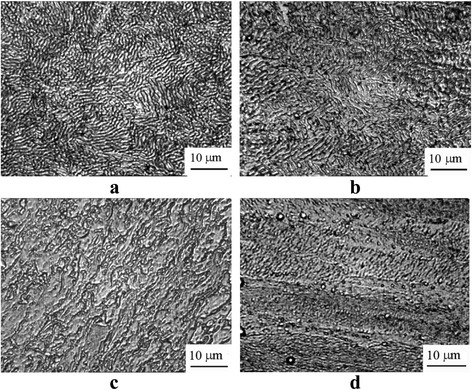
The structure of the Al-0.6%Sc-0.3%Ta alloy after deformation: HE + 4ECAH, *e*_Σ_ = 5.07, cross-section (**a**); longitudinal section (**b**); HE + 4ECAH + HE, *e*_Σ_ = 6.53, cross-section (**c**); longitudinal section (**d**)

The specimens were rotated by 90° during each pass of ECAH; thus, turbulences were observed in the cross-section domains of localized deformation in the Al-0.65Sc-0.3%Ta alloy rod after HE + 4ECAH (*e*_Σ_ = 5.07) (Fig. 3a). Domains characterized by localized deformation bands of diverse curvature were present in the longitudinal section of the sample (Fig. 3b). After further hydroextrusion (HE + 4ECAH + HE, *e*_Σ_ = 6.53) its structure was coarsened, the fine structure of the deformation domain disappeared and a unidirectional structure was formed (Fig. 3c, d).

**Fig. 4:**
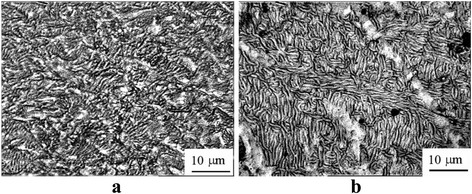
Changes in the deformation structure of the Al-0.3%Sc alloy after annealing: the initial deformation HE + 5ECAH + HE, *e*_Σ_ = 6.8 (**a**); the deformation *e*_Σ_ = 6.8 + isochronal annealing at 300 °C (*τ* = 2 h) + 400 °C (*τ* = 2 h) + 450 °C (*τ* = 2 h) (**b**)

The morphology of domains of localized deformation depends both on the deformation type and strain as well as on the composition of the alloys. So after deformation HE + 4ECAH + HE, *e*_Σ_ = 6.8, domains of localized deformation of hypoeutectic Al-0.3%Sc alloy were sectors (Fig. 4a), as opposed to domain morphology of the Al-0.6%Sc-0.3%Ta alloy after a similar deformation (Fig. 3a). A twisted fiber structure was observed after annealing these alloys (Fig. 4b).

**Fig. 5:**
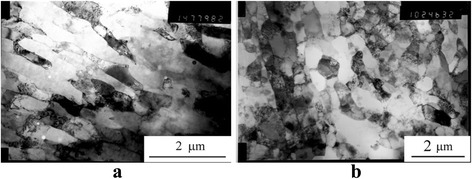
The TEM structure of the cross-section of the rod after SPD: Al-0.6%Sc-0.3%Ta HE + 2ECAH (**a**), Al-0.6%Sc-0.6%Ta HE + 4ECAH (**b**)

The TEM study showed that all experimental alloys after ECAH were characterized with the segregation of the material into active and inactive zones that formed a banded structure. In the active zones, the deformation was accompanied by the formation of dislocations, low-angle, and high-angle boundaries. Such deformation processes were absent or much less pronounced in the inactive zones (Fig. 5). The average period of the alternation of these zones (~0.2–0.8 μm) was the same as the modulation period of domains of localized deformation observed in the metallographic study. Electron-diffraction analysis revealed that the single crystal pattern in the initial state was replaced by a polycrystalline one. The number of small crystal grains increased with increasing the Sc and Ta content.

### The Effect of the SPD Type and Corresponding Accumulated Strain on the Alloy Texture

It is known that ECAH of aluminum alloys often leads to the formation of typical face-centered cubic (FCC) shear texture with multiple orientations through A ({111}<uvw>) and B ({hkl}<110>) fibers [13–18]. Yet, the texture evolution at ECAH also depends on processing route, the number of passes, die angle, deformation mechanism (e.g., slip and twinning systems), and initial texture [19].

In this work, the effect of various types of SPD (hydroextrusion (HE), HE + ECAH, and HE + ECAH + HE) and the accumulated strain on the structure and texture of cast Al-0.6%Sc-0.3%Ta alloy was studied. Longitudinal sections of the alloy specimens after ECAH and HE were investigated.

**Fig. 6:**
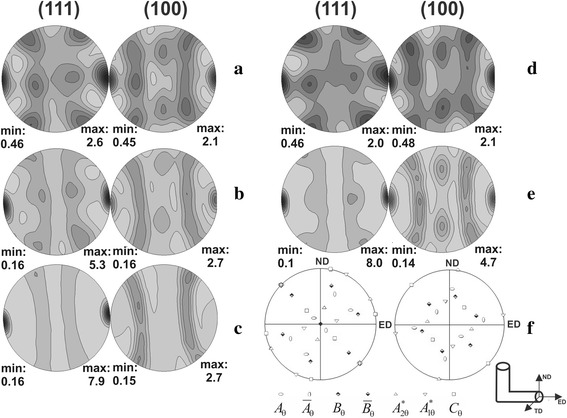
Pole figures on TD plane (**a**), (**b**), (**c**), (**d**), and (**e**) for specimens 6, 7, 8, 9, and 10, respectively, and the main ideal orientation associated with simple shear deformation of FCC materials [16] (**f**) (Table 2)

Figure 6 presents pole figures {111} and {200} on TD plane of Al-Sc-Ta alloys after different SPD treatments (Table 2).

**Table 2:** Effect of deformation type on texture, coherent domain size, and microstrains

Specimen	Deformation type	Accumulated strain *e*_Σ_	Coherent domain size *D* (nm)	Microstrains *ε* (%)	Main texture components	Pole density max *f*(*g*)
6	HE + 2ECAH	2.77	61	0.02	FCC shear texture	2.3
7	HE	2.01	200	0.13	65%<111> + 35%<100>	5.3
8	HE	2.82	–	–	<111>	7.9
9	HE + 4ECAH	4.6	100	0.05	FCC shear texture	2.0
10	HE + 2ECAH + HE	6.14	98	0.05	60%<111> + 40%<100>	8.0

It is seen that the specimen after HE followed by two passes of ECAH (specimen 6, Fig. 6a), exhibited nearly simple shear texture (Fig. 6f) with maximum pole density of 2.3. The pole figures for specimen 7 were more symmetrical as compared to those for specimen 6 after HE (Fig. 6b). The distribution of pole density for specimen 7 confirms the presence of two-component <111> + <100> fiber texture. It is also seen that maximum pole density (*f*(*g*) = 5.3) was higher than for specimen 6 after HE + 2ECAH. Further HE of specimen 7 resulted in the formation of a fiber <111> texture with maximum pole density *f*(*g*) = 7.9 (specimen 7, Fig. 6c).

Pole figures for specimen after four passes of ECAH (specimen 9, Fig. 6d) showed nearly simple shear texture (Fig. 6f) with maximum pole density of 2.1. Further HE of specimen 9 led to the formation of two-component <111> + <100> fiber texture with maximum pole density *f*(*g*) = 8.0 (specimen 10, Fig. 6e).

It should also be noted that the main texture components of the specimens 6 and 9 are slightly rotated around TD axis by some angle *Δθ* ^/^ = (*θ* ^/^ − *θ*) as compared to the ideal simple shear deformation in ECAH. Similar deviations from the orientations of simple shear were earlier reported for various materials after ECAH [14, 19, 20]. Such deviation may be caused by an additional plane strain compression component [14].

The following conclusions can be drawn from the comparison of the texture formation in the cases of monotonous (HE) and non-monotonous (ECAH) deformation. HE at the accumulated strain *e*_Σ_ = 2.01 led to the formation of two-component fiber texture 65% <111> + 35% <100>. The texture was altered by the fiber <111> texture with the maximum pole density of 7.9 at the increase of accumulated strain to *e*_Σ_ = 2.82. ECAH promoted the weakening of the texture due to the formation of partial fiber <110> and {111} textures, which are typical for FCC shear texture. The increase in the number of ECAH passes also slightly weakened the texture. The HE after ECAH recovered two-component fiber texture at the significant increase of pole density. Yet, microstrains in the specimens after ECAH + HE were nearly intact as compared to ECAH.

### The Effect of SPD on the Kinetics and Morphology of the Decomposition of the Cast Al-Sc-Ta Alloys

**Fig. 7:**
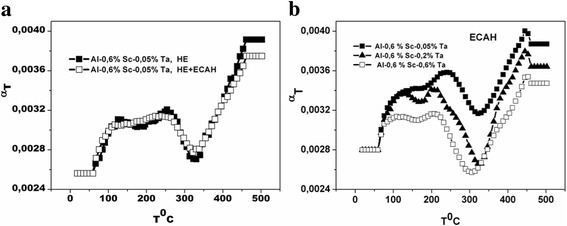
The effect of the deformation type on the change in the temperature coefficient of resistivity *α*_*T*_ = *f*(*T*) (**a**); the effect of the Ta concentration on the change in the temperature coefficient of resistivity *α*_*T*_ = *f*(*T*) after deformation HE + 5ECAH + HE *e*_Σ_ = 6,8 (**b**)

To estimate the degree of supersaturation of the alloys after SPD, the study of the temperature coefficient of resistivity, *α*_*T*_ = *f*(*T*), during continuous heating at the rate of 3°/min in the temperature range of 20–500 °C was carried out (Fig. 7).

As follows from the change of *α*_*T*_ = *f*(*T*) (Fig. 7a), neither the type nor the degree of accumulated strain affected the position and depth of the minimum. Therefore, the degree of solid solution supersaturation and the temperature range of aging remained unchanged for this alloy. The effect of SPD was more dependent on the alloy composition (Fig. 7b). The maximum supersaturation was observed for the Al-0.6%Sc-0.2%Ta alloy, as the deepest minimum was observed for it on the *α*_*T*_ = *f*(*T*) curve.

**Fig. 8:**
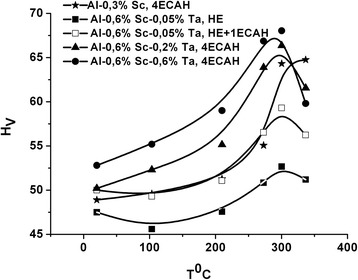
The effect of SPD on the alloy hardening during isochronal aging for 30 min in the temperature range 20–350 °C

The hardening was dependent on the degree of the accumulated strain. The increase in hardening was ~25% for the Al-0.3%Sc, Al-0.6%Sc-0.2%Ta, and Al-0.6%Sc-0.6%Ta alloys after the accumulated strain increased to *e*_Σ_ = 6.8 (Fig. 8).

**Fig. 9:**
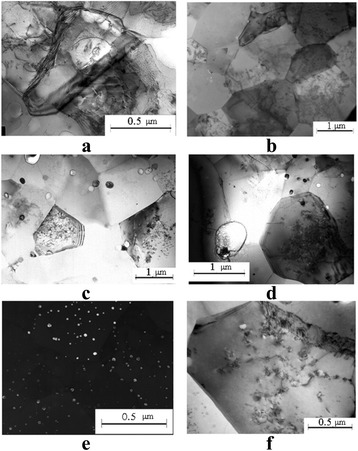
The microstructure of the alloy after SPD *e*_Σ_ = 6.8 + aging: Al-0.3%Sc, aging at 350 °C for 2 h (**a**); Al-0.3%Sc, aging at 450 °C for 2 h (**b**); Al-0.6%Sc-0.2%Ta, aging at 450 °C for 2 h (**c**); Al-0.6%Sc-0.6%Ta, aging 450 °C for 2 h (**d**); Al-0.3%Sc, aging at 450 °C for 2 h (**e**); and Al-0.6%Sc-0.6%Ta, aging at 450 °C for 2 h (**f**)

A typical electron microscopic structure after the SPD and the subsequent aging is shown in Fig. 9. A TEM study of the alloy structure after ECAH and the subsequent aging at 350 and 450 °C showed that dynamic recrystallization occurred during heating (Fig. 9a). The dynamic recrystallization was accompanied by the formation of disclinations, deformation bands, and high-angle boundaries. The dislocation structure was heterogeneous and was observed essentially within the grains which contained the bands of localized deformation (Fig. 9b). Average grain sizes increased up to ~1.5–2 μm—threefold as compared to the deformed state (Fig. 9c, d). Decomposition of supersaturated solid solution occurred through continuous and discontinuous mechanisms. In the eutectic Al-0.3%Sc alloy, the continuous decay dominated. The kinetics of decomposition slowed down significantly. Coherent particles of strengthening Al_3_Sc phase of 9 nm size were formed during aging at 450 °C for 2 h (Fig. 9e). In the alloys without SPD, these particles were semicoherent and reached the size of 30 nm. Hypereutectic alloys after SPD generally decomposed through the discontinuous mechanism at grain, subgrain boundaries, and dislocations (Fig. 9f).

**Fig. 10:**
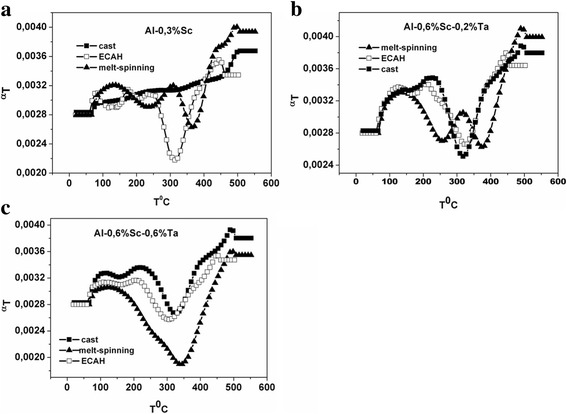
The change in *α*_*T*_ = *f*(*T*) during continuous annealing for cast alloys, after ECAH and after melt-spinning: Al-0.3%Sc alloy (**a**), Al-0.6%Sc-0.2%Ta alloy (**b**), and Al-0.6%Sc-0.6%Ta alloy (**c**)

The comparative analysis of the degree of alloy supersaturation in the cast state, after quenching from the melt by the melt-spinning method and after ECAH of the cast alloys, was carried out. As follows from the data presented in Fig. 10b, c, it was impossible to obtain the supersaturation of Sc in Al by using ECAH for hypereutectic alloys. Anomalous supersaturation was only observed after melt-spinning of these alloys. However, using ECAH at room temperature for hypoeutectic Al-0.3%Sc alloy enabled us to obtain a supersaturated solid solution without prolonged homogenization (640 °C for 10 h) and the following quenching (Fig. 10a).

**Fig. 11:**
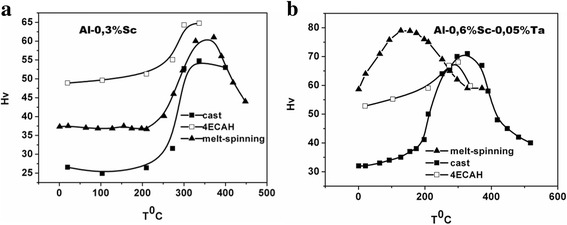
Hardness change during the isochronal aging of cast alloys, after ECAH and after melt-spinning: Al-0.3%Sc alloy (**a**) and Al-0.6%Sc-0.05%Ta alloy (**b**)

The data obtained correlated with the change in hardness during the isochronal aging (Fig. 11a, b). ECAH at room temperature for the Al-0.3%Sc alloy led to a 25% increase in hardness after isochronal aging. For hypereutectic alloys, ECAH was ineffective. The quenching of these alloys from the liquid state provided an increase in hardness by 15% due to the anomalous supersaturation.

## Conclusions

1. The SPD of Al-Sc, Al-Sc-Ti, and Al-Sc-Ta alloys leads to the reduction of internal stresses, regardless of the deformation type due to the dynamic recovery and dynamic recrystallization. Domains with the periodic structure in which the area of the deformed and non-deformed matrix alternated with a modulation period of 0.4–0.7 μm are formed. The average grain size in the alloys decreased to 0.6 μm, which resulted in the nearly twice increase of their hardness.
2. The comparison of the efficiency of monotonous (HE) and non-monotonous (ECAH) deformations of aluminum alloys during SPD at room temperature shows that ECAH as compared to HE, much more effectively promoted grain refinement and reduced the anisotropy of the rod structure.
3. In hypoeutectic Al-0.3%Sc alloys, due to the use of ECAH at the room temperature, the complete elimination of segregations was achieved without the conventional prolonged annealing. In hypereutectic Al-Sc-Ti and Al-Sc-Ta alloys, the anomalous supersaturation of the matrix by the refractory low-solubility elements was not found after SPD. The difference in the behaviors of the hypoeutectic and hypereutectic aluminum alloys was due to the presence of intermetallic particles of the crystallization origin in the latter.
4. The decomposition of supersaturated Al-Sc solid solution after SPD occurred through the mixed mechanism. The continuous decomposition mechanism was present in recrystallized grains and the discontinuous one—in nonrecrystallized grains. It was found that the precipitation density of strengthening particles significantly reduced, while the coalescence accelerated. Thus, the hardening of the alloys after SPD due to aging (dispersion mechanism) was twice weaker than the strengthening through grain refinement.

## Abbreviations

ECAH: Equal-channel angular hydroextrusion
ECAP: Equal-channel angular pressing
FCC: Face-centered cubic
HE: Hydrostatic extrusion
SPD: Severe plastic deformations
TEM: Transmission electron microscopy
